# Which patients benefit most from stereotactic body radiotherapy or surgery in medically operable non‐small cell lung cancer? An in‐depth look at patient characteristics on both sides of the debate

**DOI:** 10.1111/1759-7714.13160

**Published:** 2019-08-06

**Authors:** Gail Wan Ying Chua, Kevin Lee Min Chua

**Affiliations:** ^1^ Division of Radiation Oncology National Cancer Centre Singapore Singapore

**Keywords:** Lung cancer, stereotactic radiotherapy, surgery

## Abstract

The role of stereotactic body radiotherapy (SBRT) in early stage medically operable non‐small cell lung cancer is currently under debate. SBRT's advantage is its ability to provide high radiotherapy doses to a tumor in a short timeframe, without the risk of postoperative complications and mortality. Currently, in part due to limited prospective data comparing both treatments, international guidelines continue to recommend surgical resection as the gold standard for medically operable patients. However, not all patients possess uniform characteristics, and there is some evidence that certain subgroups of patients would benefit more from one form of treatment ‐ SBRT or surgery ‐ than the other. The aim of this review is to provide a brief summary of the evidence comparing SBRT to surgery, followed by a deeper discussion of the subgroups of patients who would benefit most from surgery: those with large tumors, centrally located tumors, increased risk of occult nodal metastases, increased risk of toxicity from radiotherapy and radioresistant histological tumor subtypes. Meanwhile, patients who could benefit most from SBRT might include elderly patients, those with reduced lung function or cardiac comorbidities, those with synchronous lung nodules, and those with specific tumor mutational status. We hope that this review will aid in the clinical decision‐making process regarding patient selection for either treatment.

## Introduction

Few innovations have had the same impact as stereotactic body radiotherapy in early stage medically inoperable non‐small cell lung cancer, with local control rates currently in the region of 85–90%.^1^ At the same time, early studies which included medically operable cohorts have reported promising results,^2^, ^3^, ^4^ hinting at clinical equipoise between lobectomy and SBRT in early stage disease. Questions have therefore arisen regarding the suitability of SBRT for medically operable patients. With a lack of phase 3 data, owing to poorly accrued trials, this dilemma continues to plague us in current clinical practice. This is exemplified in a study by Hopmans *et al*.^5^ where researchers provided 126 clinicians (including pulmonologists, thoracic surgeons and radiation oncologists) with 16 hypothetical cases of patients with stage I NSCLC. They were asked for their treatment recommendation – surgery or stereotactic body radiotherapy (SBRT), and limited consistency was observed. While recently published ASTRO guidelines^6^ have offered guidance and recommended surgery in medically operable patients, SBRT as a noninvasive therapy offers an attractive option for patients who are opposed to a surgical option. This is further accentuated by recent technological advancements in motion management and linear accelerator technology, enabling greater and cheaper access to SBRT capabilities.

## Background: SBRT

The advantage of SBRT is its ability to provide high radiotherapy doses to the tumor in a short timeframe, without risk of postoperative complications and mortality. The highly focal nature of SBRT helps to minimize radiation‐induced damage to surrounding normal lungs and organs at risk. Treatment is generally delivered in hypofractionated regimens of 3–5 fractions of 10–15 Gy per fraction, on alternate days.

The landmark trial indicating the success of SBRT in medically inoperable NSCLC patients was by Timmerman *et al*. at the Veterans Affairs Medical Center,^7^ where tumors responded to treatment in 87% of patients, with 27% showing a complete response. This showed that high radiation doses were tolerated. A subsequent phase 2 study, RTOG 0236,^1^ accrued 55 evaluable patients with T1 and T2 tumors. The three‐year local control rate was 87.2% and the DFS and OS at three years were 43.8% and 55.8%, respectively, with acceptable toxicity levels. All in all, these studies enabled SBRT to gain popularity. A Japanese multi‐institutional cohort study determined that local control and survival rates were more favorable with a BED of greater or equal to 100 Gy compared to BED <100 Gy,^8^ with BED in the range of 106–142 Gy seemingly optimal in terms of tumor control and avoiding adverse effects.^9^ With the success of SBRT in medically inoperable patients, interest has turned to SBRT as a treatment option for medically operable patients. The phase 2 RTOG 0618 study, consisting of 26 evaluable medically operable patients with T1‐T2N0M0 non‐small cell lung tumors, found that four‐year primary tumor control and local control rate were both 96%. The authors concluded that SBRT is associated with good tumor control and low morbidity in the medically operable population.^10^ A list of major prospective SBRT trials and survival data are shown in Table 1.

**Table 1:** SBRT for early stage NSCLC ‐ selected prospective trials

Study	Patient characteristics	Study size	SBRT dose	Primary endpoint	Local control	DFS	OS
Fakiris *et al*. (2009)^26^	T1/2N0M0 NSCLC up to 7 cm, medically inoperable	70 patients	60–66 Gy/3#	Local tumor control	88.1% at 3 years	81.7% at 3 years	42.7% at 3 years
Baumann *et al*. (2009)^27^	T1/2N0M0 NSCLC, medically inoperable	57 patients	45 Gy/3#	Progression‐free survival	92% at 3 years	93% at 1 year, 88% at 2 years, 88% at 3 years	86% at 1 year, 65% at 2 years, 60% at 3 years
Timmerman *et al*. (2010) RTOG 0236^1^	T1/2N0M0 NSCLC up to 5 cm, medically inoperable	55 patients	60 Gy/3#	Primary tumor control	90.6% at 3 years (within lobe) 97.6% (primary tumor control)	48.3% at 3 years	55.8% at 3 years
Ricardi *et al*. (2010)^28^	Stage I NSCLC, medically inoperable	62 patients (43 stage IA, 19 stage IB)	45 Gy/3#	Local tumor control	92.7% at 2 years and 87.8% at 3 years	79.4% at 2 years and 72.5% at 3 years (cancer‐specific survival)	69.2% at 2 years and 57.1% at 3 years
Nagata *et al*. (2015)^2^	T1N0M0 NSCLC	100 inoperable 64 operable patients	48 Gy/4#	3 year overall survival	Inoperable patients: 52.8% at 3 years Operable patients: 68.6% at 3 years	Inoperable patients: 49.8% at 3 years Operable patients: 54.5% at 3 years	Inoperable patients: 59.9% at 3 years, 42.8% at 5 years Operable patients: 76.5% at 3 years, 54.0% at 5 years
Videtic *et al*. (2015) RTOG 0915^29^ Comparing 34 Gy/1# versus*vs*. 48Gy/4#	T1/2N0M0 NSCLC, medically inoperable	84 patients	34 Gy/1# (39 patients) 48 Gy/4# (45 patients	Rates of prespecified grade 3 or higher toxicities at 1 year	34Gy/1#: 97.0% at 1 year 48 Gy/4#: 92.7% at 1 year With lower toxicity for 34 Gy group (10.3% vs. 13.3%)	34Gy/1#: 56.4% at 2 years 48 Gy/4#: 71.1% at 2 years	34 Gy/1#: 61% at 2 years 48 Gy/4#: 77.7% at 2 years
Sun *et al*., (2017)^30^	T1/2N0M0 NSCLC up to 5 cm	65 patients	50 Gy/4# (one patient received 45 Gy/4# and one received 50 Gy/3#)	Progression‐free survival	Local control: 98.5% at 1 year, 95.4% at 3 years, 91.9% at 5 years, 91.9% at 7 years Locoregional control: 93.8% at 1 year, 87.7% at 3 years, 82.6% at 5 years, 80.0% at 7 years	49.5% at 5 years and 38.2% at 7 years	55.7% at 5 years and 47.5% at 7 years
Timmerman *et al*. (2018) RTOG 0618^10^	T1/2N0M0 NSCLC up to 5 cm medically operable	26 patients	54 Gy/3#	Primary tumor control	96% at 4 years	57% at 4 years	56% at 4 years

SBRT technology has come a long way in the past few years, though there is room for continued advancements in ensuring reproducibility in terms of tumor location at breath hold, determining tumor volume and location, and shifts and rotations in the matching process.^18^

## Background: Surgery

Mirroring the developments in SBRT technology, the advent of minimally invasive surgery has meant that perioperative outcomes have improved, with the ACSOG Z0030/ alliance trial^19^ showing a five year local recurrence free survival rate of 95% for T1 tumors and 91% for T2 tumors, while five year survival was 72% for T1 tumors and 55% for T2 tumors. At present, the gold standard for surgery for early stage NSCLC is lobectomy, shown since the 1995 prospective multinstitutional trial^20^ was published comparing limited resection with lobectomy for T1N0 NSCLC where patients who underwent limited resection had an observed tripling of the local recurrence rate compared to lobectomy. The Cancer and Leukaemia Group B 39802 trial was subsequently started to evaluate the technical feasibility of VATS lobectomy and showed decreased postoperative complications compared to historical controls, establishing VATS lobectomy as the preferred option for early stage NSCLC tumors. The definition of “operability”, following recent ACCP guidelines, suggests that when postoperative FEV1 and DLCO are >60% of predicted, no further testing is required prior to resection. For either value between 30–60% predicted, further evaluation with exercise testing (e.g., stair climb/shuttle walk) should be performed.^21^

## Comparison of SBRT and surgery

For patients who present to clinic with early stage NSCLC, with few medical comorbidities and minimal symptoms, current international guidelines generally recommend surgery rather than SBRT, unless enrolled in a clinical trial. Such guidelines include those issued by the American Society for Radiation Oncology (ASTRO),^6^ the European Society for Medical Oncology (ESMO),^22^ the American Society of Clinical Oncology (ASCO),^23^ and the American College of Chest Physicians,^24^ among others. While surgery has excellent overall and disease‐free survival outcomes and offers advantages such as nodal evaluation, SBRT allows patients to avoid operative and anaesthetic risks altogether. Indeed, for patients who are not eligible for lobectomies due to high operative risk, ASTRO guidelines state that “discussions about SBRT as a potential alternative to surgery are encouraged.”^6^, ^25^ Some of the major advantages and disadvantages of the two strategies, surgery and radiotherapy, are listed in Table 2.

**Table 2:** SBRT versus surgery for early stage NSCLC: major advantages and disadvantages of each modality

SBRT	Surgery
Advantages	
Non‐invasive: avoids surgical complications, anesthetic risks	Allows full histopathologic analysis of lesion (e.g. T stage, margins)
Lower post‐treatment mortality at 30 and 90 days^11^	Facilitates pathologic lymph node staging
Able to target synchronous lung nodules where resection procedures would be extensive	Retrospective literature suggests an overall survival advantage over SBRT^12^, ^13^
Disadvantages	
Not usually utilized for tumors within 2 cm of the proximal bronchial tree due to high risk of toxicity	Not suitable for patients with poor lung function and numerous medical comorbidities
No pathological staging of lymph nodes	Post‐surgical mortality (estimates 2–4% at 30 days, 3–5% at 90 days)^11^, ^14^, ^15^
Side effects include: radiation pneumonitis, skin toxicity, odynophagia, rib fracture, pain, injury to nerves^16^	Surgical risks include infection, air leak, hemorrhage, pain, deep vein thrombosis, fistula, injury to nerves^16^
Response evaluation may be complicated by post‐radiotherapy inflammation around the tumor^17^	

Two prospective trials were instituted to evaluate surgery versus SBRT in operable patients, the STARS trial (randomized study to compare CyberKnife to surgical resection in stage I non‐small cell lung cancer) and the ROSEL trial (trial of either surgery or stereotactic radiotherapy for early stage IA lung cancer). Both closed due to poor accrual. The combined data were interpreted by Chang *et al*.^31^: 58 patients were enrolled in total, 31 randomly assigned to SBRT and 27 to surgery. Overall survival (OS) at three years was 95% in the SBRT group compared with 79% in the surgery group, HR 0.14 (0.017–1.190, *P* = 0.037). Recurrence free survival at three years was 86% in the SBRT group and 80% in the surgery group, HR 0.69 (0.21–2.29, *P* = 0.54). Ninety day mortality rates of surgery and SBRT were 4% and 0% respectively, while grade 3–4 toxicity was 44% with surgery and only 10% with SBRT. This led the authors to declare that SBRT could be an option for treating operable stage I NSCLC. Certainly in higher risk patients with more comorbidities, these considerations should be paramount in the decision‐making process.

Several retrospective analyses using propensity score matching have been performed to compare surgery to SBRT. The advantage of these is that a large number of patients are available for analyses. Overall, these have tended to favor lobectomy in terms of OS, while sublobar resections and SBRT have equivalent results. For instance, Rosen *et al*.^32^ published an analysis of the national cancer database, comparing stage I NSCLC patients free of comorbidities undergoing lobectomy (13 562 patients) compared to SBRT (1781). Propensity score matching was performed and yielded a 59% five‐year survival rate for patients who underwent lobectomy, in contrast to a 29% five‐year survival rate for those who received SBRT (*P* < 0.001). Meanwhile, Shirvani *et al*. performed an analysis of early stage NSCLC patients from the SEER database between 2003–2009. After propensity score matching, SBRT was associated with better overall survival compared to lobectomy in the first six months after diagnosis (HR 0.45; 95%CI 0.27–0.75), but this picture reversed after six months (HR 1.66; 95%CI 1.39–1.99).^12^ In a recent meta‐analysis by Wang *et al*. the authors identified two trials and seven cohort studies, using propensity score matching to compare patients in each cohort study. They concluded that the benefits of surgery were significant in terms of three‐year OS, cancer‐specific survival and recurrence‐free survival, as well as five‐year OS.^13^ In contrast, other studies such as Verstegen *et al*.^33^ and Crabtree *et al*.^34^ showed nonsignificant differences in OS after propensity score matching.

To help explore this question further, several prospective trials are now in the pipeline (Table 3). Perhaps the most sensible approach would be to discuss potential pitfalls in both modalities and identify patients who may benefit most from surgery or SBRT.

**Table 3:** Current randomised trials ‐ SBRT versus surgery in medically operable NSCLC patients

	Trial	Country	Estimated No. of participants	Phase	Estimated study completion date	Comparison
1	STABLEMATES^35^ (NCT02468024)	USA	272	III	December 2024	This will compare sublobar resection to SBRT in high risk peripheral tumors
2	POSTLIV trial^36^ (NCT01753414)	China	76	II	January 2026	This will compare radical resection to SBRT in peripheral tumors
3	VALOR Veterans Affairs study^37^ (NCT02984761)	USA	670	N/A	September 2027	This will compare lobectomy or segmentectomy to SBRT in central and peripheral tumors

The recent SABRTOOTH Trial (UK)^38^ failed to meet recruitment targets and a large RCT was deemed not to be feasible.

## Factors favoring surgery

### Large tumors

One group of patients who are likely to derive greater benefit from surgery are those with large tumors. It has been shown that for SBRT patients, local control and OS rates decrease as tumor size increases. For instance, in a retrospective analysis, Dunlap *et al*.^39^ reported that the median recurrence free survival for T1 tumors was 30.6 months after SBRT treatment while that for T2 tumors was 20.5 months, and median OS was 20 months and 16.7 months for T1 and T2 tumors respectively. Similar results were obtained by Shamp *et al*.^40^ Meanwhile, analysis by Allibhai of 185 patients who received SBRT showed that tumor size was associated with regional failure (*P* = 0.011) and distal failure (*P* = 0.021), as well as poorer overall survival (*P* = 0.001) and disease‐free survival (*P* = 0.001).^41^

Gross tumor volume and planning target volume, which increased with increasing size of tumor, were also significantly associated with grade 2 or worse radiation pneumonitis.^41^ Compounding this potential toxicity of SBRT, it also appears that higher SBRT doses may be required to achieve adequate local control in T2 tumors. For instance, in the analysis of Davis *et al*., local control was associated with higher BED_10_ (>105 gy) for T2 tumors, but not in T1 tumors at a median follow‐up of 17 months.^42^ All in all, this makes SBRT a less attractive option for larger tumors.

It should be noted that for patients who underwent surgery, the overall survival rate for those with T2 tumors is also less than that for T1 tumors. In an analysis by Nonaka *et al*.,^43^ five‐year OS for those with T2N0 tumors was 65% while that for T1N0 tumors was 85%. However, unlike toxicity of radiotherapy, which increases with increasing tumor size and PTV, morbidity of lobectomy in stage I tumors is less dependent on tumor size than other factors such as age and other comorbidities, or lower FEV1.^44^

### Centrally‐located tumors

Tumors located too closely (within 2 cm) to central structures such as trachea, bronchial tree or oesophagus result in higher doses to these OARs when delivering SBRT. In an analysis done by Timmerman's group, where 60–66 Gy total was delivered to the tumor, 83% of the patients with peripheral lung tumors had two years freedom from severe (grade 3–5) toxicity while only 54% of the patients with central lung tumors were spared.^45^ Currently, studies have shown reduced toxicity when lower doses per fraction are used, with JROSG 10–1^46^ showing the maximum tolerance dose to be 60 Gy in eight fractions, and RTOG 0813 reporting the maximum tolerance dose to be 60 Gy in 5 fractions, with a 7.2% probability of experiencing a dose‐limiting‐toxicity.^47^ However, it is not disputed that peripherally located tumors that are more distant from OARs have the potential to receive higher radiation doses with less risk of side effects.

### Risk of occult nodal metastases

The above two factors: larger tumor size and centrally located tumors, have both been shown to correspond to a higher incidence of occult nodal metastases. Overall, 15–20% of patients with early stage NSCLC have occult nodal metastases on surgical pathologic review,^48^ but not all stage I tumors are created equal in this respect. Indeed, Seok *et al*.^49^ calculated rates of lymph node metastases by tumor size in 413 patients with tumors of 3 cm or less who underwent lymph node dissection. A total of 75 patients were postoperatively found to have positive nodes, with the largest group as expected being found in those with tumors of 26–30 mm (25/53 patients). In contrast, only 10/178 patients with tumor size 2 cm or less had nodal metastases detected postoperatively. In a retrospective analysis of 894 patients, Koike *et al*.^50^ also showed that preoperative tumor size of greater or equal to 2.0 cm was an independent predictor of mediastinal nodal metastasis, and such patients could be candidates for mediastinal node assessment by invasive modalities.

In a further effort to refine risk of nodal metastases by location of tumor, Bao *et al*.^51^ found that non‐upper lobe NSCLC was a predictor of N1 or N2 node involvement. Meanwhile Ketchedjian *et al*.^52^ analysed peripheral and central tumors, defined as those visualised within the inner third of the lung field, and determined that centrally located tumors had as high as a 50% risk of lymph node metastases. Indeed, for T1 tumors, central location was an even stronger prediction of lymph node metastases than tumor size.

Park *et al*.^53^ concluded that SUVmax and metabolic total volume were significant risk factors for occult lymph node metastases in patients with small NSCLC tumors. Upon doing a ROC analysis, the optimal cut‐off values were 3.250 (sensitivity 83.3%, specificity 60%), and 3.055 (sensitivity 75.0%, specificity 67.8%) for SUVmax and metabolic total volume respectively. This is useful information as patients above the cutoff should be strongly recommended to go for pathologic nodal evaluation. To ensure increased accuracy of nodal staging, the upcoming VALOR trial includes mandatory pathologic assessment of any suspicious nodes >10 mm with SUV > 2.5 on PET CT, and the STABLEMATES trial has similar requirements.

The significance of occult nodal metastases cannot be underestimated. Patients who undergo SBRT without pathological nodal staging are taking the risk that occult nodal metastases may go undetected, thereby depriving the patient of potentially life extending chemotherapy. Indeed, in Rosen's analysis,^32^ only 6% of the SBRT patients had a pathologic assessment of lymph nodes. Meanwhile, Paravati *et al*. noted that PET‐CT staged NSCLC frequently underestimates true pathological stage,^54^ and in Crabtree's analysis, final pathology upstaged 35% (161/462) of surgery patients.^34^

A significant incidence of occult nodal metastases could explain the observation in several comparative studies that survival curves favor radiation over surgery early on (due to the issue of perioperative and postoperative mortality), but then cross between 12–36 months.^55^, ^56^ The early survival advantage of SBRT may thus be “offset” by distal recurrence – indeed in RTOG 0236, Timmerman et al,^57^ there was a relatively high risk of disseminated failure of 22.1% despite good local regional control rates at three years.

Hence, the increased risk of lymph node metastasis that comes with larger and more central tumors, as well as presence of marginally PET avid nodes, would be factors pushing patients toward surgery rather than SBRT.

### Increased risk of toxicity from radiotherapy

A subset of patients who may benefit from surgery are those at higher risk of increased toxicity from radiotherapy. For instance, Gold *et al*.^58^ studied a group of patients with systemic scleroderma who were treated with RT. Grade 1 or 2 late toxicity reactions were noted in 12/20 patients while grade 3 or higher toxicity occurred in 4/20 patients. Hence, in patients with scleroderma, risks and benefits of RT should be carefully discussed. Meanwhile, for patients with psoriasis or vitiligo, the koebner phenomenon can occur where the skin changes occurring in those conditions can be seen at areas receiving radiotherapy.^59^ Finally, in patients who have received prior radiotherapy, late adverse effects have been reported in several studies especially after single fraction doses of >10 Gy.^60^, ^61^ Hence on balance, this group of patients may derive more benefit and less risk from surgery rather than SBRT.

### Radioresistant histological tumor subtypes

Woody *et al*. analysed the response of different histological subtypes of NSCLC treated with SBRT. On multivariate analysis, squamous histological subtype (HR 2.4, *P* = 0.008) was the strongest predictor of local failure, with a three year cumulative rate of local failure of 18.9% versus 8.7% for adenocarcinoma and 4.1% for not‐otherwise‐specified.^62^ Meanwhile, Mak *et al*. genotyped lung SBRT patients for KRAS mutations and found that in patients with KRAS mutant tumors, there was significantly lower tumor control (67% vs. 96%) at one year.^63^ Finally, an analysis was done that found that low miR‐29c levels correlated with shorter relapse‐free survival of non‐small cell lung carcinoma patients treated with radiotherapy, due to increased cell survival and reduced apoptotic response.^64^ In contrast, the specific histological subtype of tumor tended to matter less in surgical cases. For instance, in a study of post surgical patients with NSCLC, while patients with SCC tended to present with larger tumors,^65^ five‐year survival rates were comparable to those with adenocarcinomas.

## Equivocal – favoring neither surgery nor SBRT

### Pre‐existing interstitial lung disease (ILD)

It should be noted that for NSCLC patients in general, whether they undergo surgery or SBRT, those with interstitial lung disease have poorer prognosis.^66^ Indeed, postoperatively, the incidence of pneumonia (acute or exacerbation of disease) was higher in the interstitial lung disease group, while the five year OS was half that of the non‐ILD group. It appears that ILD is also a poor predictor of survival and radiation toxicity for SBRT patients as well. Ueki *et al*. recorded significantly worse incidences of radiation pneumonitis (grade 2 or 3) in ILD versus non‐ILD patients, and the three year overall survival tended to be worse in ILD patients (53.8% versus 70.8%).^67^ Hence for this group of patients, the decision to choose between SBRT or surgery is not an easy one and careful discussion of such cases at a multidisciplinary board of specialists may be advisable.

## Factors favoring SBRT

### Elderly patients

Traditionally, surgeons are more hesitant to offer radical operations to elderly patients. Indeed as patients' ages increase, rates of comorbidities such as diabetes and hypertension are elevated as well, resulting in poorer surgical outcomes. Retrospective analyses have shown that patients are more likely to be offered sublobar resections than lobectomy which also has worse outcomes for tumor control.^68^ Meanwhile, SBRT has been known to be superior to no treatment in the “elderly” population, defined as age 70 or over. In an analysis of 3147 patients from the national cancer database,^69^ multivariate analysis revealed improved overall survival with SBRT compared with observation. In a retrospective analysis of 58 “very elderly” SBRT patients aged 80 or older (median 84.9), cancer specific survival rates were 73% at two years. As expected, KPS of more or equal to 75 was associated with improved outcomes.

Most interestingly, in an Amsterdam Cancer Registry Study^70^ of 875 elderly patients (age 75 and above), comparing surgery to RT to no treatment across different time periods as SBRT became more widely available, an improvement in OS was confined to RT patients whereas no significant survival improvements were seen in the other groups. This confirms the utility of SBRT in this population. In contrast, for patients who underwent surgery, in a study of 338 patients older than age 70, it was shown that a significant predictor of morbidity by multivariate analysis is age (odds ratio of 1.09 a year), as well as thoracotomy as a surgical approach. Operative mortality in this group of patients was 3.8% and morbidity was 47%, on the higher end compared to the general population (generally in the range of 1–4% at 30 days and 2–6% at 90 days following lobectomies for NSCLC.^56^, ^71^

The 2014 recommendations of the EORTC Elderly task force^72^ state that surgical treatment should not be denied to elderly patients just on the basis of chronological age, but limited resections and omission of systematic mediastinal lymphadenectomy can be considered on the basis of retrospective data. In an analysis of quality of life after lobectomy for patients less than versus greater/equal to 70 years, physical functioning remained below baseline in the older group of patients at 6 and 12 months.^73^ No equivalent stratification of QOL in elderly/young patients has been carried out in SBRT patients, but it should be noted that overall QOL seems good after SBRT in general. Among the 22 patients in the ROSEL trial, SBRT was associated with better global health status and lower indirect costs of productivity loss. This fits in with other systematic reviews^32^, ^74^ reporting few clinically significant changes in HRQOL scores after SBRT, whereas analysis of surgical patients showed increased dyspnea and fatigue persisting up to two years after surgery.^75^ Hence, on balance, SBRT may be a better option than surgery for the medically operable older patient.

### Patients with reduced lung function/cardiac comorbidities

For patients with reduced lung function tests scores or increased risk of cardiac complications, SBRT may present lower risks than surgery. “Thorascore”^76^ is a well validated tool that includes nine variables and predicts the risk of perioperative mortality. Thoracic revised cardiac risk index (RCRI) is a validated tool providing four parameters (pneumonectomy, previous ischemic heart disease, previous stroke or transient ischemic attack, creatinine >2 mg/dL) that are used to categorise patients into risk categories.^77^ Meanwhile, research has shown that in patients with preoperative FEV1 less than 35% predicted, 36% of surgical patients (lobectomies/wedge resections/pneumonectomies) had one or more complications within 30 days postoperatively, for example prolonged air leaks requiring a chest tube, pneumonias, and additional oxygen dependence.^78^ This underscores the not‐insignificant risk of carrying out surgical resections in this group of patients.

In contrast, a number of studies, for example RTOG 0236,^79^ have shown that baseline PFT did not predict pulmonary toxicity following SBRT, nor did they predict overall survival. For these borderline operative patients, especially those with peripherally located tumors where large doses of radiation can be delivered with relatively low toxicity to OARs,^80^ SBRT may be a good choice.

### Patients with synchronous lung nodules

Meanwhile, for patients who present with synchronous lung nodules in the ipsilateral or contralateral lobe where a curative surgical procedure would be extensive, SBRT has shown relatively good results. For instance, Owen *et al*.^81^ analysed 63 subjects with 128 metasynchronous and synchronous lung nodules treated with SBRT at the Mayo clinic between 2006 and 2012. A total of 18 had prior high dose EBRT to mediastinum or chest. With a median follow‐up of 12.6 months, median SBRT specific OS and PFS were 35.7 months and 10.7 months respectively. About half the patients experienced acute toxicity but this was mostly grade 1 or 2. This report demonstrated the feasibiliy of SBRT to synchronous lung nodules. Furthermore, several studies have shown feasibility of lower dose SBRT for recurrent lung cancer,^61^, ^82^, ^83^ with local control rates of up to 96% at one year and freedom from distant progression rate at 74% at one year and 65% at two years.^82^

### Patients with specific mutational status of tumors

Patients with certain tumor mutations have better outcomes after SBRT. For example, in an analysis by Blumenfeld *et al*., there was a trend towards improved PFS for EGFR mutation positive patients after SBRT, 25.4 months versus 16.7 months, and KRAS negative patients (17.8 months vs. 9.5 months). There appeared to be no difference in toxicity between patients with or without these mutations.^84^ Such studies could potentially identify a population of patients with better outcomes from SBRT.

Meanwhile, it should be noted that there are potentially immunogenic effects from SBRT. Studies have identified histologic features such as micropapillary predominant or solid with mucin‐predominant subtypes or certain gene expression profiles as higher risk.^85^, ^86^ For those borderline operable patients who opt for SBRT, these patients may be good candidates for treatment intensification with immunotherapy post SBRT. In comparison, the TRACERx cohort found that intertumor heterogeneity was associated with a higher occurrence of chromosome instability and thereby an increased risk of recurrence or death following surgery.^87^ Hence, more genomic sequencing of tumors can identify patients at higher risk after surgery or those who would benefit from treatment intensification after SBRT.

## Conclusion

While waiting for the results of upcoming trials, upfront recommendation of SBRT as an option for operable patients has not yet entered international guidelines. However, this option should be discussed with borderline operable patients in view of the benefits of avoiding higher surgical mortality and morbidity. In certain cases, especially elderly patients with noncentrally located tumors and worse lung function tests scores, the use of SBRT may be more strongly recommended. However, nodal staging is paramount: patients should be counselled that there is a possibility of PET scans missing occult positive nodes and pathological staging may well be the gold standard, providing critical information for guidance of adjuvant therapy. As more medically operable patients pursue SBRT as an option, we should keep in mind the additional possibility of surgically salvageable locoregional SBRT failures.

## Disclosure

The authors declare that they have no potential conflicts of interest, financial interests, relationships and affiliations relevant to the subject of their manuscript.
